# Chronic urticaria: new management options

**DOI:** 10.1186/1939-4551-7-31

**Published:** 2014-11-05

**Authors:** Paul A Greenberger

**Affiliations:** Division of Allergy-Immunology, Department of Medicine, Northwestern University Feinberg School of Medicine, Chicago, Illinois, 676 N. St. Clair Street, # 14108, 60611 Chicago, IL USA

**Keywords:** Urticaria, Chronic, Vasculitis, Antihistamine, H_1_ receptor, Omalizumab, Immunosuppressive

## Abstract

Chronic urticaria is defined as episodic or daily hives lasting for at least 6 weeks and impairs quality of life. Two main subtypes include chronic idiopathic (spontaneous) urticaria and inducible (physical) urticaria, but some patients have urticarial vasculitis. “Autoimmune chronic urticaria” implies the presence of histamine releasing or mast cell activating autoantibodies to IgE or FcϵRI, the high affinity receptor on mast cells and basophils. In patients not readily controlled with labeled dosages of second generation H_1_ receptor antagonists (antihistamines), there is evidence for reduction of urticaria using up to 4 fold increases in labeled dosages. The biologic modifier, omalizumab, helps to reduce lesions of chronic urticaria within 1–2 weeks.

**Electronic supplementary material:**

The online version of this article (doi:10.1186/1939-4551-7-31) contains supplementary material, which is available to authorized users.

## Introduction

The lifetime prevalence of chronic urticaria, defined as episodic or daily hives lasting for 6 weeks, occurs in approximately 1.8% of the adult population with a period prevalence (past 12 months) of 0.6 to 0.8% [1–3]. Chronic urticaria occurs in 0.1-0.3% of children [4]. Besides reducing quality of life and causing absenteeism from school and work [5], the duration of chronic urticaria in adults has been reported to be as follows: 6–12 weeks in 52.8%, 3–6 months in 18.5%, 7–12 months in 9.4%, 1–5 years in 8.7% and over 5 years in 11.3% [3]. For perspective, the lifetime prevalence of acute urticaria is 8-20% [1–3].

### Histology of chronic urticaria

**Figure 1:**
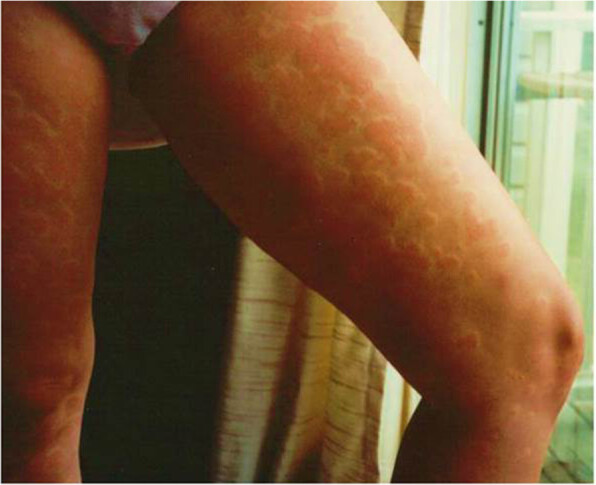
**There are plaque like erythematous lesions on the legs in a woman with H**_**1**_ **receptor antagonist resistant chronic urticaria.**

Histologic examination of lesional biopsies of patients with chronic urticaria may demonstrate distinct findings that include presence of mononuclear cells (CD4+ Th1 and Th2 lymphocytes), eosinophils, neutrophils, both eosinophils and neutrophils, basophils, mast cells (also increased in non-lesional skin), and activated macrophages [6–10]. Some biopies show edema with little or no cellular infiltrate whereas others show “perivasculitis” as there is a mononuclear infiltrate that doesn’t damage the vessel wall [7, 10] However, there may be leukocytoclastic vasculitis (cellular infiltrates present with *damage* of the vessel wall, nuclear debris, extravasation of red blood cells) despite the phenotype being an urticarial lesion that doesn’t leave residual pigment or ecchymosis [7, 10]. Lesions of chronic idiopathic urticaria are illustrated in Figure 1.

### Terminology

Chronic idiopathic urticaria, which is synonymous with chronic spontaneous urticaria, is a sub-type of chronic urticaria [2, 11, 12]. Other subtypes of chronic urticaria include the physical urticarias, “autoimmune chronic urticaria” and urticarial vasculitis. Physical urticaria (s) may coexist with chronic idiopathic (spontaneous) urticaria. The European Academy of Allergy and Clinical Immunology/Global Allergy and Asthma European Network/European Dermatology Foundation/World Allergy Organization (EAACI/GA^2^LEN/EDF/WAO) guideline [11] and the World Allergy Organization [2] make a designation of inducible urticarias (dematographic, cold by contact, delayed pressure, heat by contact, solar, aquagenic, cholinergic, contact, and vibratory). “Autoimmune” chronic urticaria implies presence of histamine releasing or mast cell activating autoantibodies to IgE or FcϵRI and is a subtype of chronic idiopathic (spontaneous) urticaria [2, 11, 12].

### Approach to the patient

In planning treatments, it is helpful to consider some unfavorable prognostic factors listed in Table 1[2, 4, 13–21]. The list primarily is based on studies of adults; physical urticaria is recognized as an unfavorable prognostic factor in children [4]. If a skin biopsy is available, it may be informative but often patients with anti-histamine resistant chronic urticaria do not have underlying urticarial vasculitis. The medical record should list the treatments (and their dosages) that have been tried, extent of reduction of pruritus, hives and angioedema, and what any untoward reactions were. The patient’s mental outlook should be assessed regarding chronic urticaria and its treatment. Some patients may doubt that a physician or healthcare professional can be helpful because of the persistence of chronic urticaria or have become nihilistic about the benefit-risk ratio of any new or previously untried treatment. Will you be able to create a partnership with the patient with shared goals for treatments and their monitoring?

**Table 1:** **Factors associated with longer duration or more difficult to treat chronic urticaria**

Factor	Comment
Failure of a single labeled dose of an H_1_receptor blocker to control chronic urticaria	Explore quality of life
Long duration (6 months or more) at time of presentation	
Angioedema	Up to 40% of patients
Physical Urticaria	Inquire about and test where indicated
Autoimmunity diseases/test results*	
Positive autologous serum or plasma intradermal skin test (some studies)	Use upmost caution with sera and plasma
Serum IgG anti-IgE or IgG anti-FcϵRI	
Hypertension	
Subclinical activation of the extrinsic coagulation pathway (Prothrombin fragments detected) or evidence of fibrinolysis (D-Dimer > 500 ng/mL)	
Basophil activation (CD203c+)	

*Applies to adults but not children for thyroid pathology/autoantibodies.

References for Table 1: [2, 4, 13–21].

### New treatment approaches as options for persisting, troublesome chronic urticaria

#### Biologic therapy

Omalizumab is effective in anti-histamine resistant chronic urticaria [22–28]. In contrast to treatment of persistent severe asthma where a patient may need to be assessed after 4–6 months of administration of omalizumab, reductions of pruritus and urticarial lesions occur within a 1 week of a single subcutaneous injection of 150 or 300 mg [22]. The severity and duration of chronic urticaria in the study subjects are illustrated by the means of 4.3 medications used and duration of lesions being 6.8 years [22]. The subjects were assessed according to an itch-severity scoring system where the highest score of 21 represented the most symptoms and impact. The baseline score (mean of 14) was compared to the last week of a 12 week active interval [22]. The placebo arm subjects took 1 second generation H_1_ receptor antagonist they had used prior to beginning the omalizumab-placebo with diphenhydramine rescue. The reduction in itch/severity score was 36% for the placebo subjects compared with 70% for active treatment with omalizumab 300 mg [22]. Thus, the number needed to treat (NNT) to benefit 1 patient is calculated as 1/absolute benefit increase or

$$\begin{array}{l} \mathrm{NNT}\;=\;1/\mathrm{experimental}\;\mathrm{result}\;\mathrm{rate}‒\mathrm{control}\;\mathrm{rate}\\ \;\mathrm{expressed}\;\mathrm{as}\;\mathrm{decimals}\;\mathrm{and}\;\mathrm{an}\;\mathrm{absolute}\\ \;\mathrm{number}\mathrm{or}\;1/0.70‒0.36\;=\;1/0.34\;\mathrm{or}\;2.9\ldots \\ \;\mathrm{an}\;\mathrm{extremely}\;\mathrm{impressive}\;\mathrm{result}\text{.} \end{array}$$

Omalizumab is approved for use in the U.S. for chronic idiopathic urticaria that is not controlled by H_1_ receptor antagonists for patients ages 12 years and older. The dosage is either 150 mg or 300 mg subcutaneously every 4 weeks. No new safety issues have been identified in treatment of patients with chronic urticaria which is reassuring. The rapid response may be a reflection of the 1) binding of omalizumab to free IgE antibodies, which occurs within a few hours of administration, that reduces the binding of IgE to the high affinity receptor FcϵRI on basophils and mast cells, and 2) downregulation of the expression of FcϵRI on whole blood basophils (within 2 weeks) and mast cells (within 8 weeks) [22]. If pharmacologic effects can be extrapolated from experiments in patients with allergic rhinitis, omalizumab has been associated with both a reduction in the allergen induced wheal size and recruitment of eosinophils into the late phase skin reaction [29]. Eosinophils are present in some patients with chronic urticaria, and activation of eosinophils in lesional skin has been demonstrated by staining for major basic protein in extracellular tissue [30]. In that major basic protein can activate mast cells [31], perhaps the anti-eosinophil effect of omalizumab is another mechanism for the reduction in lesions of chronic idiopathic (spontaneous) urticaria in some patients.

The duration of treatment remains to be established. When study patients were observed for an additional 20 weeks after the third and last injection of omalizumab at 8 weeks, there was a gradual return of pruritus and urticaria by week 20, the data suggesting a treatment duration of about 4 weeks with subsequent loss of efficacy. This finding suggests the need for longer term treatment for some patients. While patients with persistent moderate and severe asthma may discontinue treatment with omalizumab, it is usually because of lack of benefit not ontoward effects. Similar to other medications or interventions, the decision to continue omalizumab for chronic urticaria should include assessing therapeutic benefit and any untoward effects.

### Higher dosages of H_1_ receptor antagonists

Up to 4 times the labeled adult dosages of second generation H_1_ receptor antagonists, levocetirizine and desloratadine, have been shown to reduce symptoms in about 75% of patients with chronic idiopathic urticaria (including patients with concomitant physical urticaria) [32]. The 80 randomized patients with “difficult to treat” chronic urticaria included 58 (72.5%) patients who had received oral corticosteroids within the previous 3 weeks. The study patients had not achieved control with both first and/or second generation H_1_ receptor antagonists. The study design was randomized, blinded, with crossover active treatment of either levocetirizine or desloratadine, both starting at 5 mg. At 1 week intervals, the dosage of H_1_ receptor antagonist was increased to 10 mg then 20 mg if control had not been achieved. If subjects became symptom and urticaria free for 3 days (“success”), they didn’t continue to the crossover arm of the study. The study results included the following observations: [1] doubling the dosage to 10 mg was effective with both active treatments; [2] the initial success rate (increasing to 20 mg if needed) was superior with levocetirizine (22/40 subjects) compared with desloratadine (I2/37 subjects); [3] when symptomatic subjects were switched to the alternative treatment arm, therapeutic benefit occurred with levocetirizine but not desloratadine. For example, there were 7/25 subjects not achieving success with desloratadine 20 mg who became symptom free with levocetirizine 20 mg [32]. Alternatively, 0/18 subjects who had not reached success with levocetirizine 20 mg improved with desoloratadine 20 mg [32]; [4] somnolence was either unchanged from baseline or lower with both active treatments. The WAO Scientific and Clinical Issues Council and EAACI/GA^2^LEN/EDF/WAO guidelines suggest that instead of using oral corticosteroids as second-line treatment for patients with chronic urticaria, higher doses of second generation H_1_ receptor antagonists should be tried [2, 11].

### Older drugs with effectiveness for persisting, troubling chronic urticaria

#### Tricyclic antidepressants

The tricyclic antidepressant, doxepin, has been studied in 2 double blind, controlled trials [33, 34]. While doxepin has been administered for at least 30 years, it remains a potent H_1_ (and H_2_ receptor) receptor antagonist and is effective in some patients without either intolerable or any perceived drowsiness. In a study of 50 patients, doxepin 10 mg three times daily was compared with diphenhydramine 25 mg three times daily [33]. “Total clearing of the pruritus and urticarial lesions occurred in 43% of the patients while receiving doxepin and in only 5% while receiving diphenhydramine” [33]. And in another study in 16 adults, doxepin was superior to placebo and reduced the cutaneous wheals produced by histamine and codeine [34]. Anti-cholinergic side effects such as constipation and dry mouth may occur in addition to sedation. However, doxepin (and other tricyclic antidepressants such as nortriptyline) may be beneficial in difficult to treat chronic urticaria.

### Leukotriene receptor antagonists

Because intradermal injections of very small dosages of LTD_4_ cause wheal/erythema reactions [35, 36], the leukotriene receptor antagonists zafirlukast [37] and montelukast [38, 39] have been tested in patients with chronic idiopathic urticaria. In a 2 arm, placebo-controlled trial, the addition of zafirlukast 20 mg twice daily to cetirizine 10 mg daily resulted in a “modest but significant” reduction in a visual analogue scale when assessed over a 3 week period compared with cetirizine monotherapy [37]. In retrospect, those patients who had a positive autologous serum skin test response were more likely to respond to montelukast as “add-on” therapy [37]. In a double-blind, crossover, placebo-controlled study, there was no difference between montelukast, 10 mg, and placebo (including in patients with concomitant aspirin intolerance) as add-on therapy [39]. A systematic review in 2009 concluded that “montelukast might be effective in chronic urticaria associated with aspirin (ASA) or food additive hypersensitivity or with autoreactivity to intradermal serum injection (ASST) when taken with an antihistamine but not in mild or moderate chronic idiopathic urticaria (urticaria without any possible secondary causes…)” [40]. The literature suggests that if a response to a leukotriene receptor antagonist is likely, it occurs over the first 3 weeks. Thus, a patient could be tried for 3–4 weeks and if no symptomatic response occurs, the leukotriene receptor antagonist would be discontinued. It is of interest that leukotriene receptor antagonists have been reported to be effective in some types of physical urticaria such as primary cold urticaria, delayed pressure urticaria and dermatographism [40].

### Immunosuppressive drugs

The immunosuppressive drugs may be therapeutic as monotherapy for patients with uncontrolled chronic urticaria. There may be a clear-cut response to immunosuppressive drugs in the initial 1–4 weeks of therapy. Some patients respond after 3–5 months of treatment. Benefit-risk considerations should be assessed, and patients must be monitored for clinical harm and laboratory abnormalities. Cyclosporine [41–45], tacrolimus [46], mycophenolate mofetil [47, 48], methotrexate [49], azathioprine [50] and mizoribine [51] have been found effective in some patients with refractory, typically prednisone-dependent chronic urticaria. There are various reviews of the treatment choices when patients have failed multiple other therapies [52–56]. The daily dosages of cyclosporine initially were 5 mg/kg but to avoid hypertension and loss of renal function (often reversible), lower dosages have been utilized such as 1.5-2.5 mg/kg daily [41]. The patient monitors blood pressure twice a week and renal function is checked every 2 weeks at first. If the serum creatinine increases 30%, the dosage of cyclosporine is reduced. If the creatinine doesn’t return to baseline in 2 more weeks, (after a month of increase), the decision can be made to discontinue treatment [41]. The daily dosage of tacrolimus was reported as high as with 0.05-0.07 mg/kg twice daily for 4 weeks then reduced by ½ for 6 weeks [46]. Eventually the dosage was 1 mg daily. Because of side effects (abdominal pain, diarrhea, headache, etc.), this author starts with 5 mg daily for adults to determine tolerability and safety. Khan has suggested beginning at 1 mg twice daily [52]. Mycophenolate mofetil, which doesn’t cause renal impairment but can increase the risk for infections, has a starting dosage of 1000 mg twice daily [52]. Azathioprine can cause acute abdominal pain, nausea, arthralgias, abnormal liver function tests and cytopenias and also may be effective as monotherapy. This author initiates therapy in adults with 100 mg daily. Laboratory tests should be obtained every 2 weeks for the first 2 months then at a lesser interval if there is a response to azathioprine.

### Miscellaneous agents

Colchicine [57–59], dapsone [60–62] and sulfasalazine [63, 64] have anti-inflammatory effects that may contribute to reduction in the frequency and severity of urticarial lesions in treatment-resistant chronic urticaria. These agents have specific adverse effects such as diarrhea for colchicine, hemolysis and methemoglobinemia (even in glucose 6 phosphate dehydrogenase sufficient patients) for dapsone, and gastrointestinal symptoms, headache, rash, leukopenia and elevated liver function tests for sulfasalazine. Most experience is from retrospective reviews. Starting dosages in adults are as follows: colchicine 0.6 mg daily for a week then twice daily [57]; 25-100 mg daily for dapsone; and 500 mg daily increasing weekly to 2000 mg daily for sulfasalazine [64].

In that elevated concentrations of D-dimer reflect activation of the external pathway of the coagulation system and evidence of fibrinolysis, patients with treatment-resistant chronic urticaria received a low molecular weight heparin, nadroparin (11,400 IU) daily and oral tranexamic acid as an inhibitor of fibrinolysis [65]. There was “marked improvement” within 2 weeks in 5/8 patients [65]. The concentration of D-dimer declined in responders and non-responders. It is suspected that tissue factor, which activates the coagulation cascade, is derived from eosinophils in chronic urticaria [66]. Warfarin has been reported as a possible treatment in a small double-blind, controlled, crossover trial in which the International Normalized Ratio (INR) was between 2.0 and 2.5 [67]. In patients who improved, there was no reduction in the response to intradermal injections of histamine or the mast cell activator, compound 48/80 [67]. Besides inhibiting thrombin and reducing synthesis of protein C and the vitamin K-dependent factors (prothrombin and VII, IX, and X), warfarin reduces generation of kinin, activation of complement, and down-regulates vascular adhesion molecules.

Approved for Leishmaniasis because of its anti-trypanosomatid parasite activity and recognized as a drug with antineoplastic effects, the protein kinase B inhibitor, miltefosine, was reported to reduce the urticaria activity score in antihistamine resistant patients (UAS7) over a 4 week period compared with placebo [68]. The intensity of pruritus was not lessened. Side effects include vomiting, diarrhea, elevated liver function tests and increases in serum creatinine. How miltefosine will be used in difficult to control chronic urticaria remains to be determined.

### Summary

Chronic urticaria impairs quality of life and in about ½ of patients doesn’t respond readily to labeled dosages of a single H_1_ receptor antagonist. While increasing the dosage up to 4 fold of a second generation agent, levocetirizine [32] or desloratadine [32] has been shown to be useful in some patients, this is roughly equivalent to using potent and long lasting first generation H_1_ receptor antagonists (wherein hydroxyzine 25 mg is comparable to cetirizine 10 mg) [54]. The opportunity to treat with omalizumab for chronic idiopathic (spontaneous) urticaria provides a safe approach that has resulted in reduction in pruritus and number of hives within a week of the first subcutaneous dosage [22]. The number of patients to treat to benefit 1 patient with omalizumab is 2.9, a very favorable (low) number. It will be important to determine if longer term treatment can cause disease remission.

## Authors’ original submitted files for images

Below are the links to the authors’ original submitted files for images. Authors’ original file for figure 1
