# Supplementary figures and images for: Chronic urticaria: new management options
Source: World Allergy Organ J. 2014 Nov 5;7(1):31. doi: 10.1186/1939-4551-7-31 (PMC4223736; doi:10.1186/1939-4551-7-31)

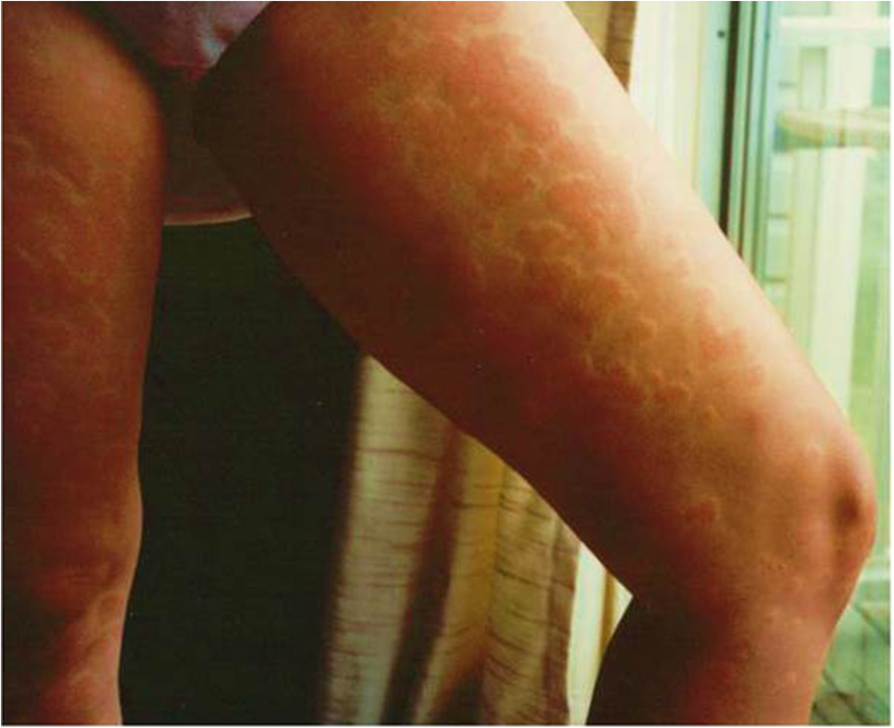

Supplement: Supplementary file 1 — Authors’ original file for figure 1 [file 40413_2014_70_MOESM1_ESM.tiff]
